# Laparoscopic Management of Intra-Abdominal Testis: 5-Year Single-Centre Experience—A Retrospective Descriptive Study

**DOI:** 10.1155/2012/878509

**Published:** 2012-02-14

**Authors:** Tariq O. Abbas, Ahmed Hayati, Adel Ismail, Mansour Ali

**Affiliations:** ^1^Pediatric Surgery Department, Hamad General Hospital, Doha 3050, Qatar; ^2^Urology Department, Hamad General Hospital, Doha 3050, Qatar

## Abstract

*Background*. Undescended testis is one of the most common urological problems in children, affecting about 1% of boys at age of 1 year. Of these, about 20% have a nonpalpable testis with a very high probability that the testis is absent. This may have a significant impact on the possibility of malignancy in these testes, as well as on the later fertility of these subjects. *Methods*. We retrospectively analyzed the demographic and clinical findings, as well as immediate and 6-month outcomes, in 91 patients diagnosed with impalpable undescended testes between January 2006 and December 2010. *Results*. Of the 91 patients, 9 had bilateral and 82 had unilateral impalpable testes. All 100 testes were managed laparoscopically. The largest group of intra-abdominal testes in this series, 42 testes, was entering the internal ring; in these, laparoscopic exploration and standard open orchiopexy resulted in a 66% success rate. The total success rate was 63.3%. *Conclusion*. Laparoscopy is extremely useful in both the diagnosis and treatment of impalpable testes. Objectively measured mobility of the testis towards the contralateral internal inguinal ring is an excellent intraoperative indicator for type of orchiopexy. Standardization of management may increase the success rate of orchiopexy.

## 1. Introduction

About 1-2% of boys at age of 1 year have an undescended testis (UDT); this disorder is unilateral in about 90% of individuals and bilateral in about 10% [1–3]. Almost 20% of undescended testes are nonpalpable [4]. Undescended testes are usually evaluated and managed by imaging methods and surgery, respectively [5].

Laparoscopy was first used in 1976 to locate undescended testes [6]. Surgical treatment of undescended testis has included dividing the spermatic vessels to gain additional length and bringing the testis to the scrotum while maintaining the collateral blood supply [5]. The first stage of this procedure was later modified to include laparoscopic ligation of the spermatic vessels [7].

We describe here our single-institution experience with laparoscopic management of impalpable testes in children over the last 5 years.

## 2. Methods

**Figure 1:**
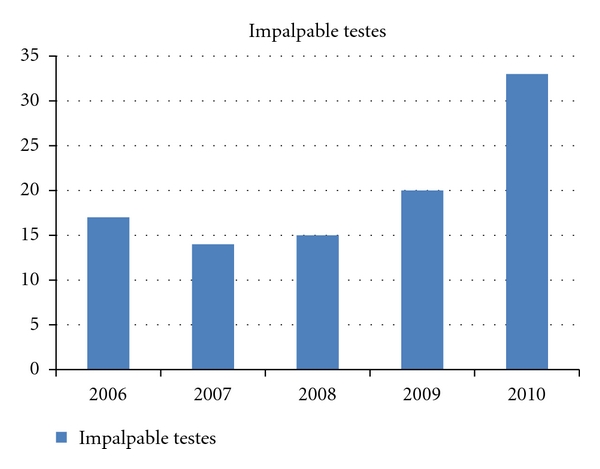
Numbers of impalpable undescended testes explored laparoscopically per year from January 2006 to December 2010.

 We retrospectively assessed the records of our institution to identify all patients below 14 years of age who underwent laparoscopy for impalpable testes between January 2006 and December 2010 (Figure 1).  We identified 91 patients, 9 with bilateral and 82 with unilateral impalpable testes (total of 100 testes) who were laparoscopically managed.

**Figure 2:**
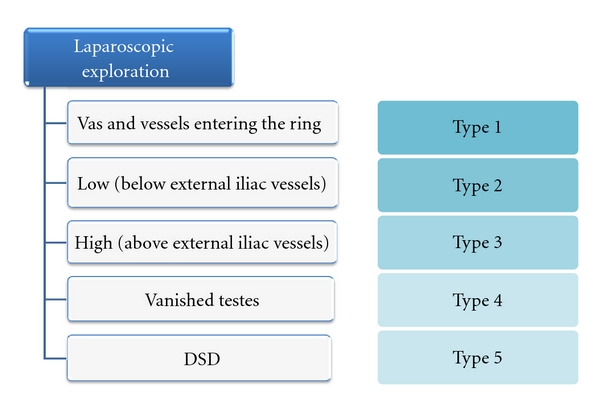
Laparoscopic findings and subsequent management of patients with undescended testes. DSD: disorders of sexual differentiation.

All patients were reexamined under anesthesia to confirm that the testes were intra-abdominal. Laparoscopic exploration was performed by inserting a 5 mm port supraumbilically using closed techniques and using a 5 mm 0 camera. Secondary 2-3 mm ports were placed under direct vision if required, and a 2 mm atraumatic grasper was used. We attempted to identify the testes, testicular vessels, vas deferens, and whether the internal inguinal rings (IIRs) were open or closed. Laparoscopic findings were classified according to the patterns of these structures and used to determine subsequent management (Figure 2).

A “high” position of the testis was defined as being above the external iliac vessels; orchiopexy for these patients consisted of a two-stage Fowler-Stephens procedure. A “low” intra-abdominal testis was usually managed by one-stage laparoscopic orchiopexy. Orchidectomy was performed on an atrophic testis accompanied by a contralateral normal testis. All patients were routinely followedup at our outpatient clinic. A “successful” procedure was defined as a testis palpable in the scrotum and of similar or increased size.

Factors evaluated included patient age at operation, side of the impalpable testis, clinical and laparoscopic findings, operative intervention, and outcomes. 

## 3. Results

**Figure 3:**
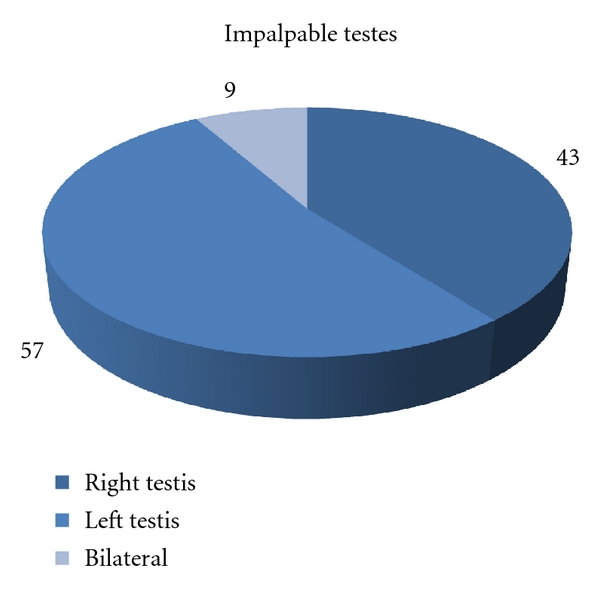
Distribution of impalpable testes according to the side affected.

We identified 91 patients, 9 with bilateral and 82 with unilateral impalpable testes, between January 2006 and December 2010, for a total of 100 testes. There was a trend of increasingly performed cases over that period (Figure 1). Mean patient age at the time of surgical intervention was 19 + (interquartile range [IQR], 12–36) months. The sides of impalpable testes are shown in (Figure 3).

Thirty-seven patients received no further treatment after laparoscopy due to absent testes. In contrast, we found that 11 intra-abdominal testes were high, above the iliac vessels, and 5 were low.

The 11 high intra-abdominal testes were managed using the two-stage Fowler-Stephens procedure. This procedure was successful for 3 testes, after a mean followup period of 3.5 months, but unsuccessful in 4; of the latter, 3 underwent orchidectomy for atrophic testes. The remaining 3 were lost to followup.

Seven “low” intra-abdominal testes were managed using the one-stage Fowler-Stephens procedure, which was successful in 1 and unsuccessful in 1; the remaining 5 were lost to followup.

In 42 testes, the vas and vessels entered the internal inguinal ring; open-standard inguinal orchiopexy was successful in 20 patients and unsuccessful in 10; the latter underwent a second orchiopexy. The remaining 12 were lost to followup Table 1.

None of these patients experienced any immediate or postoperative complications from laparoscopy. No port site hernia was detected at followup.

## 4. Discussion

Testicular descent, although not yet fully understood, takes place during two different gestational stages, occurring during intrauterine weeks 8 to 15 and 25 to 35. Failure of the first phase of descent is rarer than of the second phase and results in an intra-abdominal undescended testis [8]. 

However, cryptorchidism is one of the most common genitourinary disorders in young boys. Although the management of boys with palpable testes has been standardized, there are no formal guidelines for the management of boys with nonpalpable testes [9]. 

Laparoscopy is currently the most reliable diagnostic modality in the management of impalpable testes. It clearly shows the anatomy and provides visual information upon which a definitive decision can be based [10]. Three main laparoscopic findings are possible: intraabdominal testis, observed in 40% of patients; intra-abdominal blind-ending cord structures, observed in 15%; cord structures entering the internal inguinal ring, observed in 45% [11].

Although the right side is more frequently in undescended testes (45%) in comparison to left side (35%), we have found in our study that 57% of the patients with unilateral nonpalpable testes were in the left side while 43% in the right side.

If no testis can be visualized or the vas or vessels end blindly before the ring, a thorough laparoscopic examination should be performed, especially since gubernacular blood vessels can be mistaken for blind-ending spermatic vessels [12]. If the blind-ending vessels are not accompanied by an associated vas deferens, an ectopic testis should be suspected [13].

Despite 15 years of international research on the topic, there are no guidelines on the management of boys with nonpalpable testes [9]. If an intra-abdominal testis is normotrophic, the optimal method of performing an orchidopexy must be chosen [14–16]. For example, if the testis is located at the internal ring without looping of the vas, laparoscopic orchiopexy without division of the spermatic vessels may be performed, but the testis may not reach the bottom of the scrotum [5]. 

Routine open inguinal orchiopexies has yielded good results, as shown by testicular size and position, in patients with *type 1* testes, in which the vas and vessels enter the internal ring. In patients with *type 2*, however, where the testes are low or at the internal ring but the vas does not loop distally, we routinely test the length of the spermatic cord to determine the potential for successful setting of the testes in their hemiscrotal home. This test consists of pulling the testis towards the contralateral internal ring; if it reaches there comfortably, there is a high possibility of easy fixation. Over the 100 testes included in this study, 7 were in this category.

In *type 3* where the testes have difficulty in reaching the contralateral internal ring, laparoscopically staged Fowler-Stephen orchiopexy is the procedure of choice. We observed a success rate of 42.9%, comparable to previous findings.

We found that the total success of orchiopexy was 63.3% in line with previously reported rates (Table 2). 

In conclusion, laparoscopy is an extremely useful and safe modality for both the diagnosis and management of impalpable testes. An excellent intraoperative indicator in deciding on the type of orchiopexy is the mobility of the testis towards the contralateral internal inguinal ring.

## Figures and Tables

**Table 1:** Outcomes relative to intraoperative laparoscopic categorization of impalpable testes.

Findings	Type	Details	Number (= %)	Success numbers	No followup	Success %
Vas and vessels entering internal ring	Type 1		42	20	12	66
Testes seen	Type 2	Low (below external iliac vessels)	7	1	5	50
	Type 3	High (above external iliac vessels)	11	3	4	42.9
Vanished testis group	Type 4	Absent or rudimentary	37	N/A	N/A	N/A
Intersex patients' group	Type 5		2	2	0	100

**Table 2:** Total success rate of orchiopexy in our study and in previous studies.

Study	Number of testes	Success rate (%)
Docimo et al. (1995) [17]	80	81.3
Kirsch et al. (1998) [18]	33	97
Dhanani et al. (2004) [19]	28	100
Our study (2011)	100	63.3
